# Cure of Necrosis in the Lower Maxilla

**Published:** 1893-04

**Authors:** T. E. Lee


ARTICLE IV.

### CURE OF NECROSIS IN THE LOWER MAXILLA.

BY T. E. LEE, M. D., D. D. S.

On the 2d of April, 1890, Bridget Clancy came to my office, Dr. A., her physician, had sent her to Dr. B., a very prominent surgeon, for advice, who in turn directed her to a dentist to have teeth extracted preparatory to having a portion of the lower jaw removed.

I learned from her the following history of the case: About the 1st of June, 1889, a large swelling appeared about one inch to the left of the symphysis of the lower maxilla. The swelling was lanced by her physician, Dr. A., who, according to the girl's statement, pronounced it a disease of the blood. I found the girl to be of a tuberculous diathesis.

After the lancing by Dr. A., a fistulous opening resulted externally. This was treated by poulticing, cauterization, and internal or constitutional treatment from June, 1889, to April. 1890. She said that Dr. A. asserted the trouble could not possibly be from her teeth, as it was too low down on her chin. She was finally taken to Dr. B., who discovered that a large portion of the left half of the bone was necrosed, and said it would have to be removed. Carefully examining her mouth, I found the left lower cuspid and first bicuspid badly decayed with foul and decomposed pulps. The mucous membrane covering their fangs, and that in the immediate vicinity, was very much inflamed and punctured by a number of small ragged openings. The ragged edges were of proud flesh. A probe could be introduced into any of them to a considerable distance, without causing any pain. On the external surfaces of the chin and lower portion of the face somewhat similar conditions were seen. There was considerable inflammation and swelling. The large fistulous opening, and at least five smaller, the latter very similar to those found on the mucous membrane over the roots. The bone at these points seemed to be completely honeycombed. Still there was no evidence of a loosened sequestrum. I anæsthetized the patient, and extracted the two teeth described above, whose foul and poisonous pulps had caused all the trouble by percolating through the apices of the roots into the bone. The poor girl had been so deeply impressed by the belief that she would have to lose a part of her jaw, she actually dreamed, while under the anæsthetic, it was being removed.

I undertook the treatment of the case. I at once syringed very warm carbolized water through the teeth sockets and fistula. Very easily done, for on syringing the sockets the water came through the opening on the chin and *vice versa*. I ordered the application of flax seed meal poultice, to reduce the inflammation and swelling; had her return to the office about every third day for the warm water treatment, with which I sometimes used listerin instead of carbolic acid. The water was forced through, so as to perfectly clean all accessible parts. She was directed to bathe the parts freely in warm water each night before retiring. The largest external opening was drained by means of twisted cotton for about one week. The poultices were applied four times. In the process of healing, several flaps of callus skin were left, giving rather a ragged appearance to the scar. These were clipped off closely with scissors. After having been under treatment about four weeks, the girl met with an accident by receiving a severe blow on that portion of her chin, which no doubt somewhat retarded the progress to cure. I will state I painted the parts, both externally and on the mucous membrane, frequently with equal parts of aconit, iodin and chloroform, and gave her the following as a tonic internally:

R.—Quinia sulphatis - - ℈j.

Ferri oxalati - - - ℈ij.

Ext. nucis vomicæ - - gr. v.

M. et ft. in pil. xx.

Sig. One an hour after each meal.

At the expiration of ten weeks I discharged her cured. The fistulous opening healing prettily, only a small dimple being left as a scar, and that almost out of sight under the chin. The case had gone on nearly a year before coming to me.

The right lower cuspid and first bicuspid were also abscessed.—*Items of Interest.*

